# Critical digital ischemia and biliary cholangitis related to graft versus host disease: A case report and systematic literature review

**DOI:** 10.1097/MD.0000000000032495

**Published:** 2023-01-13

**Authors:** Cristina Hidalgo Calleja, Carlos Alberto Montilla Morales, María Dolores Sánchez González, Sonia Pastor Navarro, Marta Ibáñez Martínez, Alberto Conde Ferreiros, Lucía López Corral

**Affiliations:** a Rheumatology Department, Complejo Asistencial Universitario de Salamanca-IBSAL, Salamanca, Spain; b Rheumatology Department, Hospital Clínico Universitario de Valladolid, Spain; c Dermatology Department, Complejo Asistencial Universitario de Salamanca-IBSAL, Salamanca, Spain; d Haematology Department, Complejo Asistencial Universitario de Salamanca-IBSAL, Centro de Investigación del Cáncer-IBMCC, Salamanca, Spain.

**Keywords:** allogeneic hematopoietic cell transplant, biliary cholangitis, graft versus host disease, Raynaud phenomenon

## Abstract

Chronic graft versus host disease (cGVHD) is a systemic immune-mediated complication that occurs in approximately half of patients undergoing allogeneic hematopoietic stem cell transplantation (allo-HCT), and remains the leading cause of late morbidity and mortality. cGVHD involves a heterogeneous group of organic manifestations, many of which mimic autoimmune diseases such as scleroderma, primary biliary cholangitis, Sjögren syndrome and polymyositis.

**Patient concerns:**

A 60-years-old female with a history of allo-HCT developed *de novo* cGVHD 11 months after allo-HCT with isolated liver involvement. The patient presented with jaundice, cytolysis, cholestasis and concomitant acute digital ischemia. Liver biopsy and autoimmunity tests were performed and were found to be compatible with immune-mediated liver damage. Nailfold capillaroscopy revealed microangiopathy, characterized by avascular areas and some enlarged capillaries resembled an early systemic sclerosis pattern.

**Diagnosis:**

Biliary cholangitis-like and digital ischemia related to cGVHD.

**Interventions:**

The patient was treated with high-dose prednisone and ursodeoxycholic acid, and extracorporeal photopheresis. The patient required hospital admission for administration of intravenous prostacyclin due to refractory Raynaud syndrome.

**Outcomes:**

After 6 to 8 weeks, the patient achieved a good response, with evident clinical improvement and progressive normalization of liver function.

**Lessons:**

cGVHD is a multiorgan pathological condition, and this case emphasizes that a multidisciplinary team, including rheumatologists, should be involved in the follow-up of allo-transplant patients to ensure that the clinical complications are adequately addressed. Early intervention is critical for improving patient’ prognosis.

In addition, we performed a systemic literature review based on published case articles on hepatic cGVHD and digital ischemia published up to August 2022. To the best of our knowledge, this is the first reported case of such an association.

## 1. Introduction

Allogeneic hematopoietic stem cell transplantation (allo-HCT) is a highly specialized and complex medical procedure. In recent decades, allo-HCT has gone from being an experimental procedure to a curative treatment for many congenital and acquired malignant and nonmalignant hematological diseases, and its use is steadily increasing.^[1,2]^ The main cause of mortality after allo-HCT is relapse of the underlying disease. In addition, a high percentage of patients will have morbidity and mortality related to the transplant because of the inherent toxicity of the procedure. Of these transplant-related complications, the most important are graft versus host disease (GVHD)^[3]^ and infections.

Chronic graft versus host disease (cGVHD) is a systemic immune-mediated complication that occurs in approximately half of patients undergoing allo-HCT.^[4]^ Although it is associated with beneficial graft versus tumor effect and lower relapse rates, it remains the leading cause of late morbidity and mortality after allo-HCT.^[5]^ cGVHD involves a heterogeneous group of organic manifestations, many of which mimic autoimmune diseases such as scleroderma, primary biliary cholangitis (PBC), Sjögren syndrome and polymyositis, among others.^[6–8]^ In addition, the occurrence of Raynaud phenomenon (RP), sometimes in severe form, has been described in association with endothelial dysfunction on rare occasions.^[8]^

We present a patient with a history of allo-HCT who developed *de novo* cGVHD 11 months after allo-HCT with isolated liver involvement compatible with biliary cholangitis-like and concomitant severe digital ischemia. This case is, to our knowledge, the first case of this association to be reported.

## 2. Clinical case presentation

A 60-years-old woman was diagnosed with *T*-cell prolymphocytic leukemia in September 2020. She was treated with alemtuzumab and subsequently, in February 2021, underwent a reduced-intensity conditioning (fludarabine plus busulfan), matched peripheral blood stem cell-related transplant. Disease status at transplantation was the first partial remission. GVHD prophylaxis consisted of tacrolimus plus sirolimus plus mycophenolate. The patient achieved complete remission on day + 100 after HCT, but developed cutaneous relapse on day + 180. Disease relapse was treated by discontinuation immunosuppression, bexarotene and PUVA.

Eleven months after allo-HCT the patient presented jaundice with cytolysis and cholestasis (total bilirubin 8.7 mg/dL, direct bilirubin 6.7 mg/dL, gamma glutamyl transpeptidase 158 U/L, alkaline phosphatase 146 U/L, lactate dehydrogenase 250 U/L, aspartate aminotransferase 194 U/L, alanine aminotransferase 198 U/L). Liver biopsy and autoimmunity tests were performed and found to be compatible with immune-mediated liver damage and positive auto/allogeneic antibodies (subtype M2 antimitochondrial antibodies > 1000 U/mL) (Table 1). She was treated with 1 mg/kg of prednisone, ursodeoxycholic acid and extracorporeal photopheresis, with progressive normalization of liver function.

**Table 1:** Results of hepatic and immunological tests.

Test	Value in patient
Bilirubin	Total: 8.74 mg/dL (Reference value: 0.3–1.2 mg/dL) Conjugated: 6.7 mg/dL (n < 0.3 mg/dL)
Transaminases	Alanine transaminase: 198 U/L (Reference value: 10–49 U/L) Aspartate transaminase: 194 U/L (Reference value: ≤ 31 U/L)
Gamma-glutamyl transferase	158 U/L (Reference value: 46–116 U/L)
Alkaline phosphatase	146 U/L (Reference value 46–116 U/L)
Coagulation test	Partial thromboplastin time: 26.4 s (Control: 23.2–30.4 s) Prothrombin activity: 89% (Control: 70–120%)
Serum albumin test	2.8 g/dL (Reference value 3.4–5.0 g/dL)
Complement	C3: 76 mg/dL (Reference value: 90–180 mg/dL) C4: 11 mg/dL (Reference value: 10–40 mg/dL)
Antibodies by indirect immunofluorescence ANA pattern IF: HEp-2	Antinuclear antibodies: positive 1/160 Cytoplasmic reticulum (C21): AMAs > 1000 U/mL Granular nucleolar (AC9)
Hepatitis virus serological tests	HBsAg-negative Anti-VHBs-negative Anti-VHBc-negative Anti-VHC-negative

AMAs = antimitochondrial antibodies.

**Figure 1.**
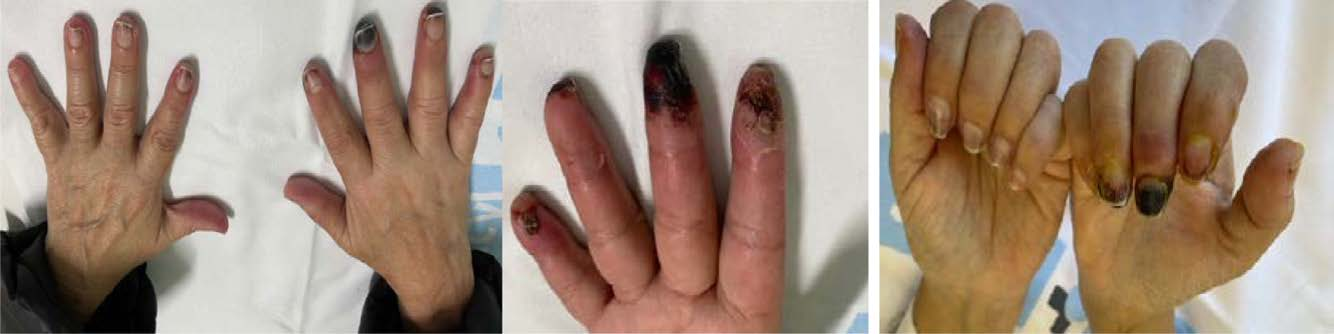
Photograph of the patient’s hands prior to prostacyclin treatment.

**Figure 2.**
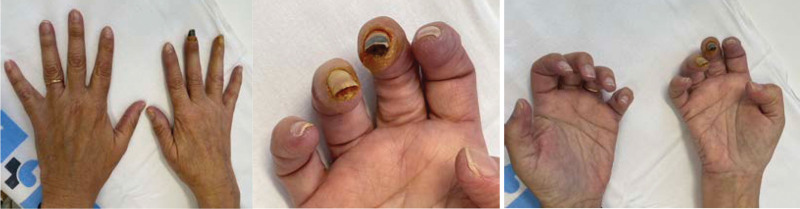
Photograph of the patient’s hands after prostacyclin treatment (2 cycles).

Concomitantly and abruptly, she started with coldness, dysesthesias, and color changes of the fingers compatible with triphasic RP. Laboratory tests detected positive antinuclear auto/alloantibodies with a titer of 1/160, the AC9 granular nucleolar pattern plus cytoplasmic reticular pattern *C*21, negative sclerosis blot, negative cryoglobulins, negative antiphospholipids, *C*3 76 mg/dL, *C*4 11 mg/dL. Nailfold capillaroscopy showed microangiopathy, characterized by avascular areas and some enlarged capillaries resembled an early systemic sclerosis pattern. No skin damage was observed. Treatment with calcium antagonists and analgesia was started but the evolution was unfavorable over a few days due to the development of critical ischemia in the distal phalanx of the third, fourth and fifth fingers and ulcerative-necrotic lesions in some of the fingers (Fig. 1). The patient required admission in the hospital and administration of intravenous infusion of prostacyclin at a dose 2 ng/kg/min over 6 hours for 5 days followed by oral sildenafil 20 mg/8 hours per day. Although the patient initially improved, with less dysesthesia and pain, 3 weeks later her condition worsened, and she developed critical ischemia that required a second 5-day cycle of intravenous prostacyclin. After 6 to 8 weeks, the patient achieved a good response with evident clinical improvement characterized by the disappearance of pain. As can be seen in Figure 2, at that time, most of the fingers were warm and had normalized vascularization, except for the right third fingertip.

## 3. Methodology and search strategy

**Figure 3.**
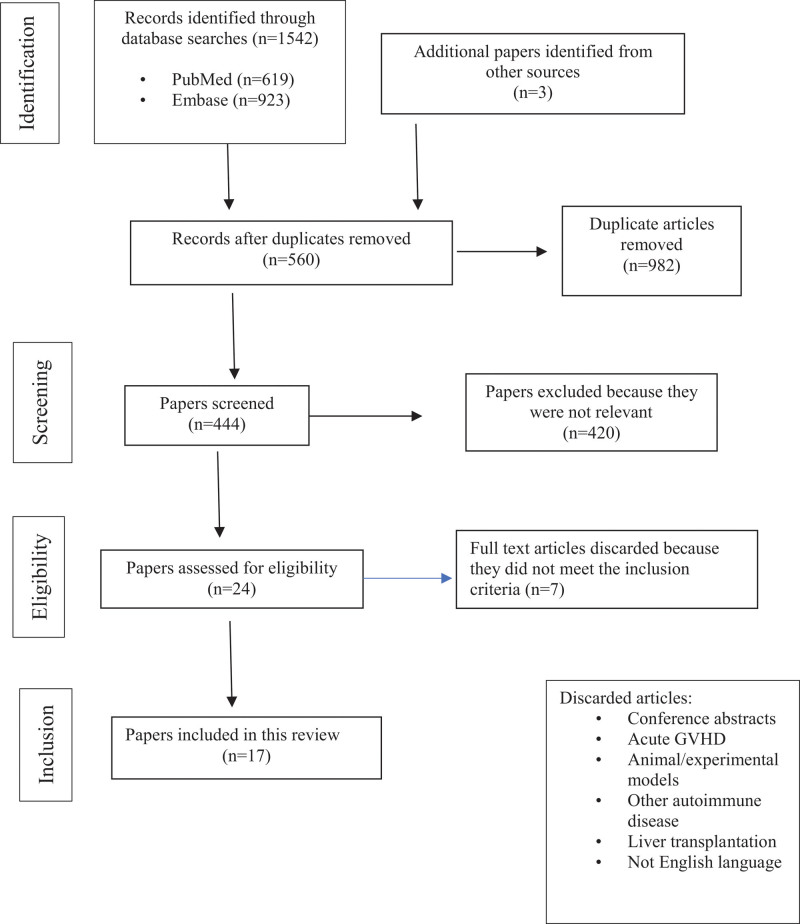
Flow-chart of selection of articles.

Subsequently, a case-based search was conducted. We performed an electronic search of the PubMed/Medline and Embase databases. We searched articles available up to August 2022, using the following search terms: `digital ischemia´ AND ´GVHD´, `Raynaud´ AND ´GVHD´, `cholangitis´ OR `biliary cirrhosis´ AND `GVHD´, ´digital ischemia´ AND ´cholangitis´ AND `GVHD´, ´Raynaud´ AND ´cholangitis´ AND ´GVHD´, ´liver´ AND ´chronic GVHD´ (Fig. 3). Our literature search retrieved 619 and 923 studies from PubMed and Embase, respectively. Articles duplicated across databases were excluded. Only publications involving humans are included in this review. Case reports published in the English language were also reviewed. All articles were further filtered by the coauthors (LLC, SPN, MIM, CMM, ACF). In case of disagreement, the papers were reevaluated and a final decision made. After excluding abstracts, acute liver GVHD, animal or experimental models, liver transplantation and other irrelevant papers, all the remaining articles, case series and case reports published before August 2022 were reviewed. Additional pertinent literature searches on specific terms, such as Raynaud disease and biliary cirrhosis, were also obtained.

Finally, 17 reports and other publications, including review papers, were selected for the case-based review.

The study was approved by the Drug Research Ethics Committee of Salamanca University Hospital and the patient provided written informed consent to publish the images in accordance with the Declaration of Helsinki.

## 4. Discussion

GVHD remains the major cause of late non-relapse death after allo-HCT.^[9]^ The pathophysiology of cGVHD is poorly understood. GVHD occurs when donor *T* cells activate and respond to HLA differences in recipient’s tissue.^[10]^ These *T* cells recognize the recipient as “non-self” and trigger a variety of immune-mediate mechanisms that directly hit the host tissues (GVHD).^[11]^ From a clinical point of view, cGVHD is a multiorgan pathological condition characterized by skin manifestations, including involvement of other organs and tissues, such as the ocular and oral mucosa, joints and fascia, lungs and liver, among others. cGVHD can occur simultaneously with acute GVHD (overlapping syndrome), after acute GVHD, or may arise *de novo*,^[12]^ as in the patient presented here. A distinctive feature of cGVHD disease is that many of its clinical and biological manifestations resemble those of an autoimmune disease, which is commonly defined as a self-directed inflammatory condition occurring in various tissues and organs, involving the innate and adaptive immune systems, and characterized by the production of several autoantibodies (aAbs).^[13]^ Indeed, patients with cGVHD may develop extensive skin scleroderma-like lesions, but organ involvement typical of systemic sclerosis (SSc), RP with capillaroscopic alterations and/or autoantibodies were rarely found. Barausse et al^[8]^ described 10 patients with scleroderma cGVHD. Only 1 patient developed RP after allo-HCT and only 1 patient exhibited a nailfold videocapillaroscopic scleroderma pattern. Except for cutaneous involvement of cGVHD, which may resemble SSc, the clinical features of the 2 diseases are quite different, suggesting that the fibrotic processes characterizing cGVHD and SSc have different etiologies and different initial pathobiological events.

PBC is another autoimmune disease, characterized by autoimmune biliary epithelial cell destruction, that leads to a chronic cholestatic liver disease, and shares clinical features with cGVHD liver injury, as in our case.^[14,15]^ Its strong autoimmune component is characterized by antimitochondrial antibodies (AMAs) and its coexistence with other autoimmune disorders, the most frequent being SSc and Sjögren syndrome.^[16,17]^

The liver is a target organ of GVHD. After day + 100, chronic hepatic GVHD is the primary cause of liver injury, occurring in about 40% of allogeneic patients.^[18]^ Liver GVHD usually presents with progressive or sudden elevation of alkaline phosphatase and gamma glutamyl transpeptidase levels, whereas liver cGVHD affecting bile ducts and resulting in severe hyperbilirubinemia occurs less frequently. In fact, hyperbilirubinemia is usually a late manifestation that coincides with development of cirrhosis and the evidence of small bile ducts destruction. The reported clinical case abruptly presented jaundice and severe hyperbilirubinemia with positive allo/autoantibodies and histopathological findings on the hepatic biopsy confirming biliary cholangitis (BC). In this setting, a biopsy may be required to establish the diagnosis of liver cGVHD.^[19,20]^and to exclude other harmful hepatic causes (viral infections, biliary obstruction, drug toxicity, nonalcoholic steatohepatitis and malignancy). Histological study is especially appropriate when the only manifestation of cGVHD is liver involvement, as was the case in the patient presented here. Diagnosis of BC was based on clinical symptoms and signs, serological tests and histological findings.^[21]^ Detection of AMAs, especially of the M2 subtype (AMA-M2), are highly sensitive and specific to PBC in clinical settings and are considered to be a serological hallmark of PBC.^[22,23]^ The histological features noted resembled those of primary cholangitis.^[8,24]^ Wakae described 12 patients with hepatic cGVHD similarity to PCB with negative AMAs but HLA DR features of PBC, suggesting that HLA DR features of PBC may also be risk factors for the onset of hepatic GVHD.^[25]^

RP is a common vasospastic condition which carries a significant burden of pain and hand-related disabilities. RP consists of an exaggerated vascular response to cold temperature or emotional stress. This phenomenon is manifested clinically by sharply demarcated color changes of the skin of the digits and may include pain resulting from low blood flow or ischemia.^[26]^ RP is a clinical diagnosis. RP is considered to be primary or idiopathic if these symptoms occur alone, without evidence of any associated disorder. In comparison, secondary RP refers to the presence of the disorder in association with a related illness, such as systemic lupus erythematosus (SLE) or SSc. In fact, many diseases and exposures are associated with secondary RP.^[27]^ The ischemia can be transient or prolonged, with complete recovery or varying levels of tissue injury. In this regard, the patient reported herein presented an acute and severe ischemia with sudden compromise of nutritional blood probably arising in the context of cGVHD. Nailfold capillaroscopy is a method commonly used to help distinguish patients with primary RP from those with secondary RP.^[28]^ Enlarged or distorted capillary loops and/or dropout or loss of loops suggests an underlying systemic rheumatic disease, or an increased likelihood of developing 1. If the enlargement is associated with loss of capillaries, then the patient is more likely to have or develop SSc.^[29]^ Our patient has not so far presented any symptoms of skin sclerosis involvement. However, several aAbs has been detected in the presented case, one of which had a nucleolar pattern described in SSc, without any specific antigen.

RP has been rarely described in patient with scleroderma related to cGVHD. Our literature review identified only 2 articles about the use of nail capillaroscopy in sclerodermoid cGVHD.^[30,31]^ To our knowledge this is the first case described in the literature of RP and BC that is related to cGVHD.

Despite the overlapping features of SSc and sclerodermatous cGVHD, some clinical and laboratory characteristics still distinguish cGVHD from SSc, like microvascular changes as evaluated by capillaroscopy, aAbs and RP. Akay et al^[30]^ studied the characteristics of nailfold capillary changes in sclerodermoid cGVHD. They found distinct nailfold capillaroscopy patterns in patients with this condition that were similar to SSc, However, their presence did not confer any special risk for any other specific clinical symptoms of the disease. A subsequent study in sclerodermatous cGVHD patients failed to show the characteristic microvascular abnormalities observed in SSc, suggesting that capillary damage does not contribute to the pathophysiology of sclerodermatous cGVHD, with the implication that capillaroscopy is unsuitable for early identification.^[31]^ However, the capillaroscopy in the case reported here showed features suggestive of early SSc (avascular areas but no megacapillaries).

## 5. Conclusion

Chronic GVHD is the leading cause of liver injury following allogeneic transplantation. However, RP has rarely been described as a complication of cGVHD. In this report, we present a patient with a history of allo-HCT who developed *de novo* cGVHD with isolated liver involvement compatible with primary biliary cholangitis-like and concomitant severe digital ischemia without any other symptoms of the sclerodermiform fenotype of cGVHD. To the best of our knowledge, this is the first reported case of such an association.

cGVHD is a multiorgan pathological condition and, this case emphasizes that a multidisciplinary team, including rheumatologists, should be involved in the follow-up of allo-transplant patients to ensure that the clinical complications are adequately addressed. The search for new biomarkers, the use of advanced imaging techniques and a multidisciplinary approach will help improve the prognosis of these patients.

## Acknowledgements

Phil Mason for English editing.

## Author contributions

**Conceptualization:** Cristina Hidalgo Calleja, Alberto Conde Ferreiros, Lucía López Corral.

**Formal analysis:** Carlos Alberto Montilla Morales, María Dolores Sánchez González, Sonia Pastor Navarro, Marta Ibáñez Martínez, Alberto Conde Ferreiros.

**Investigation:** Cristina Hidalgo Calleja, Alberto Conde Ferreiros.

**Methodology:** Cristina Hidalgo Calleja, Carlos Alberto Montilla Morales, María Dolores Sánchez González, Sonia Pastor Navarro, Marta Ibáñez Martínez, Lucía López Corral.

**Supervision:** Lucía López Corral.

**Validation:** María Dolores Sánchez González.

**Writing – original draft:** Cristina Hidalgo Calleja.

**Writing – review & editing:** Cristina Hidalgo Calleja, Lucía López Corral.
